# Clinical reliability and validity of a video-based markerless gait evaluation method

**DOI:** 10.3389/fped.2023.1331176

**Published:** 2023-12-22

**Authors:** Jincong Lin, Yongtao Wang, Jia Sha, Yi Li, Zongzhi Fan, Wei Lei, Yabo Yan

**Affiliations:** ^1^ Department of Orthopedics, Xijing Hospital, Air Force Military Medical University, Xi’an, China; ^2^ School of Telecommunications Engineering, Xidian University, Xi’an, China; ^3^ Guangzhou Institute, Xidian University, Xi’an, China; ^4^ Department of Orthopaedics, Affiliated Jinling Hospital, Medical School of Nanjing University, Nanjing, China

**Keywords:** human posture estimation algorithm, markerless gait analysis, lower limb kinematics, reliability, validity

## Abstract

**Objective:**

To explore the reliability and validity of gait parameters obtained from gait assessment system software employing a human posture estimation algorithm based on markerless videos of children walking in clinical practice.

**Methods:**

Eighteen typical developmental (TD) children and ten children with developmental dysplasia of the hip (DDH) were recruited to walk along a designated sidewalk at a comfortable walking speed. A 3-dimensional gait analysis (3D GA) and a 2-dimensional markerless (2D ML) gait evaluation system were used to extract the gait kinematics parameters twice at an interval of 2 h.

**Results:**

The two measurements of the children's kinematic gait parameters revealed no significant differences (*P* > 0.05). Intra-class correlation coefficients (ICC) were generally high (ICC >0.7), showing moderate to good relative reliability. The standard error of measurement (SEM) values of all gait parameters measured by the two walks were 1.26°–2.91°. The system software had good to excellent validity compared to the 3D GA, with ICC values between 0.835 and 0.957 and SEM values of 0.87°–1.71° for the gait parameters measured by both methods. The Bland–Altman plot analysis indicated no significant systematic errors.

**Conclusions:**

The feasibility of the markerless gait assessment method using the human posture estimation-based algorithm may provide reliable and valid gait analysis results for practical clinical applications.

## 1. Introduction

In clinical practice, abnormal gait is one of the most common symptoms in pediatric orthopedic clinics (1), accounting for approximately 35.5% of pediatric orthopedic outpatient visits (2, 3). Gait abnormality in children is a non-specific symptom that has many causes, including trauma, deformity, inflammation and tumors (3). However, most of these effects are physiological. Therefore, distinguishing between physiological and pathological gaits is challenging for doctors.

Owing to the particularity of children, outpatient doctors in clinics typically employ the visual method to make qualitative judgments, which mainly depend on their subjective experiences (4). This approach often leads to missed diagnosis of the disease, and it is impossible to give a quantitative basis for diagnosis. In addition, some doctors rely heavily on imaging tools for evaluation (5), which not only increases outpatient imaging examination rates but also impacts children's health due to radiation and other factors. At present, in some developed areas, doctors can make an accurate 3-dimensional gait analysis (3D GA) of the human gait using professional equipment (6–8). To date, this method has been applied to the diagnosis and treatment of cerebral palsy, flatfoot, and other diseases in children (9, 10). 3D GA is based on the principles of biomechanics and applies computer-aided and infrared camera technology to systematically analyze the kinematics and dynamics of gait and has good reliability and validity (11, 12). However, owing to the need for expensive equipment and professional technicians to operate it, 3D GA is currently only applied in a few medical centers with gait laboratories (13). Other shortcomings of this gait assessment technique include the need to mark subjects' joints, poor co-operation of children, difficulty, and long processing time for data collection. Therefore, there is an urgent need for a better, lower cost, more intelligent, and more reliable walking posture analysis system for children to analyze their gait for application in routine clinical practice.

With the development of human posture estimation algorithms, it is possible to use video captured with handheld devices and output by motion capture system software to assist in medical treatment (14–16). By recording a video of a subject walking, the movements in each frame of the video and the joint angles can be captured and tracked to obtain relevant gait parameters, which are then used to assist in the diagnosis and treatment of diseases. Sabo et al. (17) provided a simple gait monitoring method based on a human posture estimation algorithm for elderly people who need to be in a long-term care environment. Ouyang et al. (18) applied a human pose estimation algorithm based on the OpenPose framework to analyze the movements of patients with attention deficits before and after being treated with medication to assess drug efficacy. Similarly, based on the OpenPose system, Viswakumar et al. (19) tested the influence of different clothing or lighting conditions on joint angle measurement and found that joint angle is not easily affected by ambient light and clothing changes and has good accuracy. However, at present, the markerless gait evaluation system based on the human posture estimation algorithm is mainly used on adults (20, 21). As there are certain differences in gait between children and adults, a system suitable for adults cannot simply be applied to children. Therefore, a markerless gait evaluation system for children is needed.

The software is primarily based on existing human posture detection algorithm, Keypoints And Poses As Objects (KAPAO) (22). This new single-stage 2D multiplayer keypoint-and-pose detection method was proposed by researchers at the University of Waterloo, Canada. Its advantage is that it is not based on thermal map to estimate key points. Compared with previous algorithms, the algorithm is stable and fast. When developing this type of system software, it is important to ascertain its reliability and validity (11). However, to the best of our knowledge, current research on the reliability of this markerless gait assessment method is more applicable to adults (23–25), whereas there are few studies that explore the reliability and validity of the markerless gait assessment method for children. DDH is one of the common diseases in pediatric orthopedics. Children often have abnormal gait with limited movement of hip and knee joints. It has always been the focus of pediatric orthopaedic surgeons to pay attention to the gait changes of children with DDH. The markerless gait assessment method is expected to provide relevant data for pediatric orthopedic surgeons, but its reliability and accuracy have not been verified.

In summary, this study aimed to develop a practical clinical markerless gait evaluation software based on a human posture estimation algorithm and to verify its reliability and validity by recruiting TD children and DDH children, in order to evaluate the feasibility of this method for clinical application. The software is assumed to have good reliability and validity.

## 2. Methods

### 2.1. Subjects

The study subjects were healthy volunteers, and the inclusion criteria were as follows: (1) children aged 3–11 years, (2) can walk 10 m independently and smoothly. Eighteen TD children (males: females = 8:10) and ten DDH children (males: females = 4:6) were included in the current study based on the inclusion criteria. Table 1 shows participants' characteristics and demographics. All parents of the children provided informed consent for the testing, and the study was conducted in accordance with the Declaration of Helsinki. The protocol was reviewed and approved by the institutional review board of the First Affiliated Hospital of the Air Force Medical University.

**Table 1:** Demographic characteristics of the participants.

Characteristics	TD Mean (SD)	DDH Mean (SD)
Age, years	6.22 (2.02)	6.08 (2.22)
Body height, cm	118.47 (15.46)	119.88 (15.90)
Body mass, kg	24.23 (8.86)	22.56 (6.51)

### 2.2. Experimental setup

**Figure 1:**
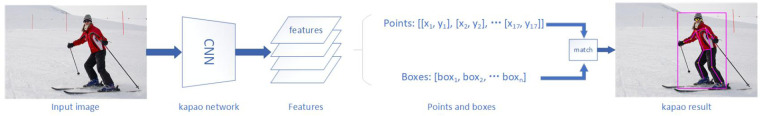
Example output for pose estimation based on KAPAO.

**Figure 2:**
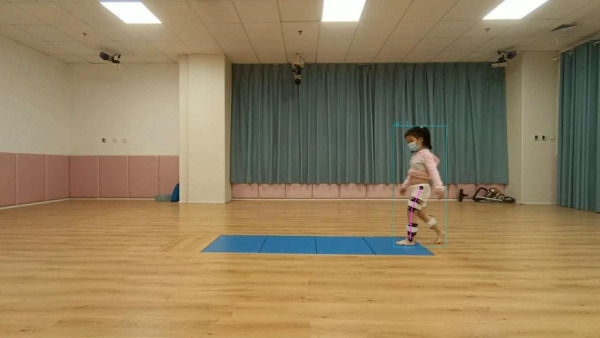
Image of the output of the markerless gait evaluation system.

The markerless gait evaluation system consisted mainly of a smartphone camera (1,920 × 1,080 pixels at 30 frames/s) and video analysis software at the computer terminal. The software is primarily based on existing human posture detection algorithm, Keypoints And Poses As Objects (KAPAO) (22). KAPAO is a top-down approach for pose estimation that enhances the YOLOv5 object detection algorithm by adding 17 keypoints outputs in the detection head. This allows it to simultaneously detect the human body's position and the positions of 17 corresponding keypoints. Figure 1 demonstrates an example of KAPAO predicting human keypoints. To detect human bounding boxes and keypoints, KAPAO modifies the YOLOv5 output to adapt it to the task of human pose estimation. Initially, the input image undergoes feature extraction through a backbone network. Subsequently, four feature layers extracted from the backbone network are passed to Feature Pyramid Network (FPN) and Path Aggregation Network (PAN) for multi-scale fusion (26). Next, KAPAO decodes bounding boxes and their keypoints. The KAPAO model encodes human bounding boxes and keypoints together. Each output box consists of a bounding box and 17 keypoints, with each keypoint treated as a class of bounding box. If the class of the bounding box corresponds to a human, the model decodes the human and its keypoints. However, if the class corresponds to keypoints, the model decodes the center of the box as a keypoint. Finally, a matching algorithm is employed to merge human bounding boxes and keypoints, resulting in the final output (Figure 2).

**Figure 3:**
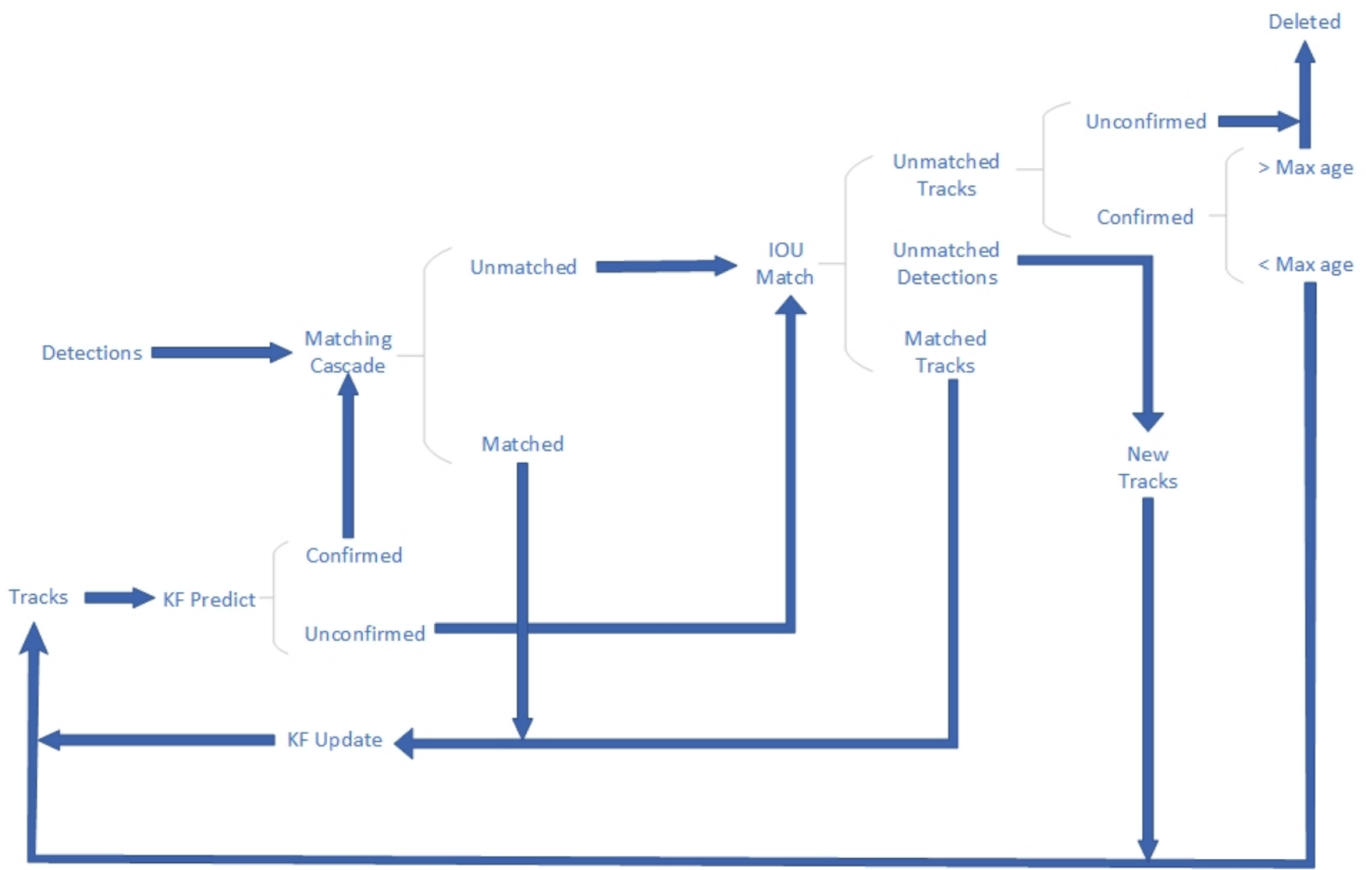
Deep sort pipeline.

In the task of child gait recognition, it is often necessary for adults to provide guidance to encourage children's cooperation during testing. Consequently, other individuals may appear in the background of the recorded video. Identifying the correct individuals from the video is an urgent issue to be addressed. DeepSORT (27) is a tracking algorithm evolved from SORT (28), which assigns a unique label to each person appearing in the video and tracks their trajectories. For tracking individuals, the DeepSORT algorithm employs a Convolutional Neural Network (CNN) to extract features from the human bodies and uses a Kalman filter to predict their trajectories. Figure 3 displays the structure of the DeepSORT algorithm. Firstly, the DeepSORT algorithm collects all the detection boxes from the current frame's object detection output and matches them with the predicted boxes from the Kalman filter output using a cascading matching algorithm. Successfully matched detection boxes are forwarded to the next Kalman filter cycle. Detection boxes that could not be matched in the cascading matching are further matched based on Intersection over Union (IoU). This process results in three scenarios: (1) If a detection box successfully matches a predicted box, the detection box is updated and sent to the Kalman filter for predicting the next round of predicted boxes. (2) If a detection box does not match any predicted box, and if an appropriate predicted box is not found, it signifies the appearance of a new trajectory in the image. A new trajectory is then created and sent to the Kalman filter for prediction. (3) If a predicted box does not match any detection box, two possibilities exist. If the predicted box is in a non-deterministic state, it is considered a false positive due to detection algorithm errors, and the trajectory is discarded. However, if the predicted box is in a deterministic state, it is possible that the trajectory is obscured by objects or has exited the camera's view. Therefore, a threshold is set, and if a re-match occurs within the threshold, it implies the trajectory was obscured. If no re-match occurs beyond the threshold, it suggests the trajectory has exited the camera's view, and the trajectory is directly discarded. Note: Non-deterministic states transition to deterministic states after successful matching in multiple rounds.

The software mainly consists of three modules: (1) a video acquisition module: obtains the vide (the video contains gait images of children or adults walking, and the video does not restrict shooting scenes and shooting equipment); (2) a human posture detection module: detects the human body detection frame of each human body object in the video and the human body keypoints in the frame using the preset human body posture detection algorithm; and (3) a human body tracking module: matches the human body detection frame of each human body object in the video frame by frame and generates the continuous gait feature of the human body object according to the human body key points in the matched human body detection frame (Supplementary Video S1). The two-dimensional coordinates of the hip, knee, and ankle joints of the human object in the video are extracted, and the required kinematic angle is calculated according to these coordinates. The flexion and extension angles of the hip joint in the sagittal plane are calculated by the angle between the long axis of the thigh and the reference line perpendicular to the ground: the flexion and straightening angles are positive and negative, respectively. To calculate the flexion and extension angles of the knee joint in the sagittal plane, the extension line of the long axis of the thigh is used as the reference line. The angle is the angle between the reference line and the long tibial axis. Once again, the flexion and straightening angles are positive, and negative, respectively.

A smartphone with an instrumented 3D GA with plug-in-gait model was used to record each child's gait. Measurements were conducted using an eight-camera motion analysis system at a sampling frequency of 100 Hz. (MX-T 20S; Vicon; Oxford, UK). A total of 38 reflective spherical markers were affixed to the body surface anatomy of the participants. For example: lateral condyle of femur, medial condyle of femur, anterior superior iliac spine, anterior inferior iliac spine and the head of the fifth metatarsal, etc. The joint angle was calculated using visual 3D software.

### 2.3. Data collection

First, we set up a video capture device [mainly composed of smartphones installed on tripods at a height of 75 cm (19) in a 3D GA room to record the subject's sagittal plane. Specifically, the smartphone recording in the sagittal plane was parallel to the subject's sidewalk, and the distance from the sidewalk was 250 cm to capture the 8-meter-long sidewalk in the field of view of the camera. The camera parameters were set to 1,920 × 1,080 pixel images at 30 frames/s, and each subject wore shorts and a t-shirt and walked barefoot on the sidewalk at their usual speed (Supplementary Video S2). Two complete walking tests were then performed. The interval between the two walking tests was approximately 2 h. The subjects could walk on the sidewalk before the test to familiarize themselves with the experimental process.

### 2.4. Data analysis

3D GA and markerless motion capture software were used to extract the kinematic angle of each frame from the video. Three complete gait cycles for each participant were used in the analysis. The gait parameters in the gait cycle were calculated and analyzed using Excel (: maximum flexion angle of the hip joint in the sagittal plane, maximum extension angle of the hip joint, minimum flexion angle of the hip and knee joints, maximum flexion angle of the knee joint, and range of motion (ROM) of the joint.

SPSS 26.0 (IBM, Armonk, United States) was used for data processing and statistical analyses. Validity and reliability included both relative and absolute values. First, a paired sample *t*-test was used to compare the difference between the two measurements, and the relative reliability was analyzed using the intra-class correlation coefficient (ICC). An ICC value less than 0.5, between 0.50 and 0.75, between 0.75 and.09, and above 0.9 indicates poor, moderate, good, and excellent reliability, respectively (29). The absolute reliability was analyzed using the standard error of measurement (SEM). $\mathrm{SEM}\;=\;\mathrm{SD}\times \sqrt{1-\mathrm{ICC}}$ (SD is the standard deviation of the average value of the two tests) (30). The error provided by SEM was consistent with the unit of measurement, and an error between 2° and 5° was considered acceptable (31). To detect whether there was a systematic error, the difference between the two measurement results was analyzed using the Origin 2022 Bland–Altman diagram, and the 95% consistency limit (limit of agreement) was marked on the Bland–Altman diagram.

## 3. Results

### 3.1. Reliability verification

There was no significant differences in the gait parameters between the two measurements using the markerless gait evaluation system software (*P* > 0.05). In terms of relative reliability, the ICC values of all gait parameters ranged from 0.736 to 0.894. Specifically, the ICC value of the maximum flexion angle of the hip joint of TD children was 0.736, indicating moderate reliability, and the ICC values of the other gait parameters were greater than 0.75, indicating good reliability. In terms of absolute reliability, the SEM values of all gait parameters were less than 3°, and the reliability was acceptable (Tables 2, 3).

**Table 2:** Measurement of sagittal mean angle and test-retest reliability in DDH children by two-dimensional markerless (2DML) method.

Gait variables (°)	First processing Mean (SD)	Second processing Mean (SD)	*t*-value	*p*-value	ICC	95% ICC	SEM
Maximum hip extension	−12.99 (4.18)	−13.16 (5.26)	1.300	0.647	0.791	0.522–0.917	2.14
Maximum hip flexion	30.36 (4.51)	30.77 (3.40)	−0.282	0.264	0.736	0.423–0.892	2.03
Hip ROM	43.35 (6.21)	43.93 (6.75)	−0.980	0.566	0.793	0.530–0.917	2.91
Minimum knee flexion	7.91 (3.81)	8.31 (4.06)	0.214	0.317	0.894	0.743–0.959	1.26
Maximum knee flexion	60.24 (3.36)	60.46 (3.06)	−1.085	0.692	0.752	0.449–0.900	1.58
Knee ROM	51.50 (4.94)	52.15 (5.23)	−1.242	0.784	0.831	0.609–0.932	2.07

First processing: gait parameters tested by markerless motion capture system software for the first time.

Second processing: gait parameters tested by markerless motion capture system software for the second time.

**Table 3:** Measurement of sagittal mean angle and test-retest reliability in DDH children by two-dimensional markerless (2DML) method.

Gait variables (°)	First processing Mean (SD)	Second processing Mean (SD)	*t*-value	*p*-value	ICC	95% ICC	SEM
Maximum hip extension	−9.04 (1.75)	−9.25 (1.17)	0.652	0.53	0.762	0.296–0.935	0.71
Maximum hip flexion	26.97 (2.75)	27.61 (2.28)	−1.186	0.266	0.768	0.310–0.937	1.19
Hip ROM	36.01 (3.79)	36.87 (2.69)	−1.362	0.206	0.817	0.423–0.951	1.38
Minimum knee flexion	10.56 (2.09)	10.35 (1.65)	0.659	0.526	0.858	0.528–0.963	0.69
Maximum knee flexion	58.20 (2.95)	57.89 (1.60)	0.616	0.553	0.774	0.323–0.939	1.10
Knee ROM	47.64 (2.84)	47.44 (1.82)	0.383	0.711	0.775	0.287–0.934	1.10

### 3.2. Validity verification

**Figure 4:**
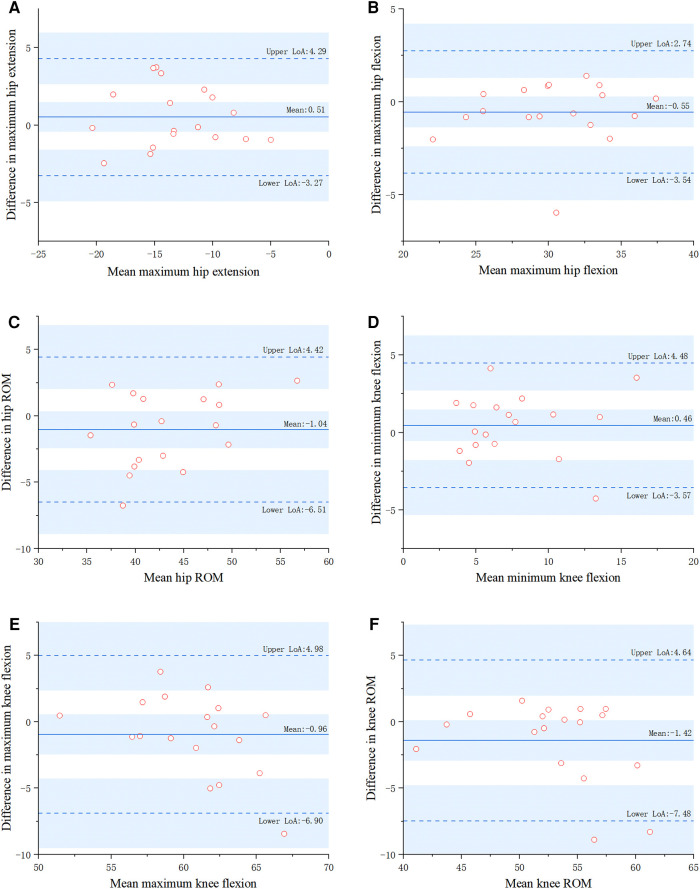
Two methods for parametric measurements of TD children using Bland–Altman plots (2D ML, 2-dimensional markerless; 3D GA, 3-dimensional gait analysis).

**Figure 5:**
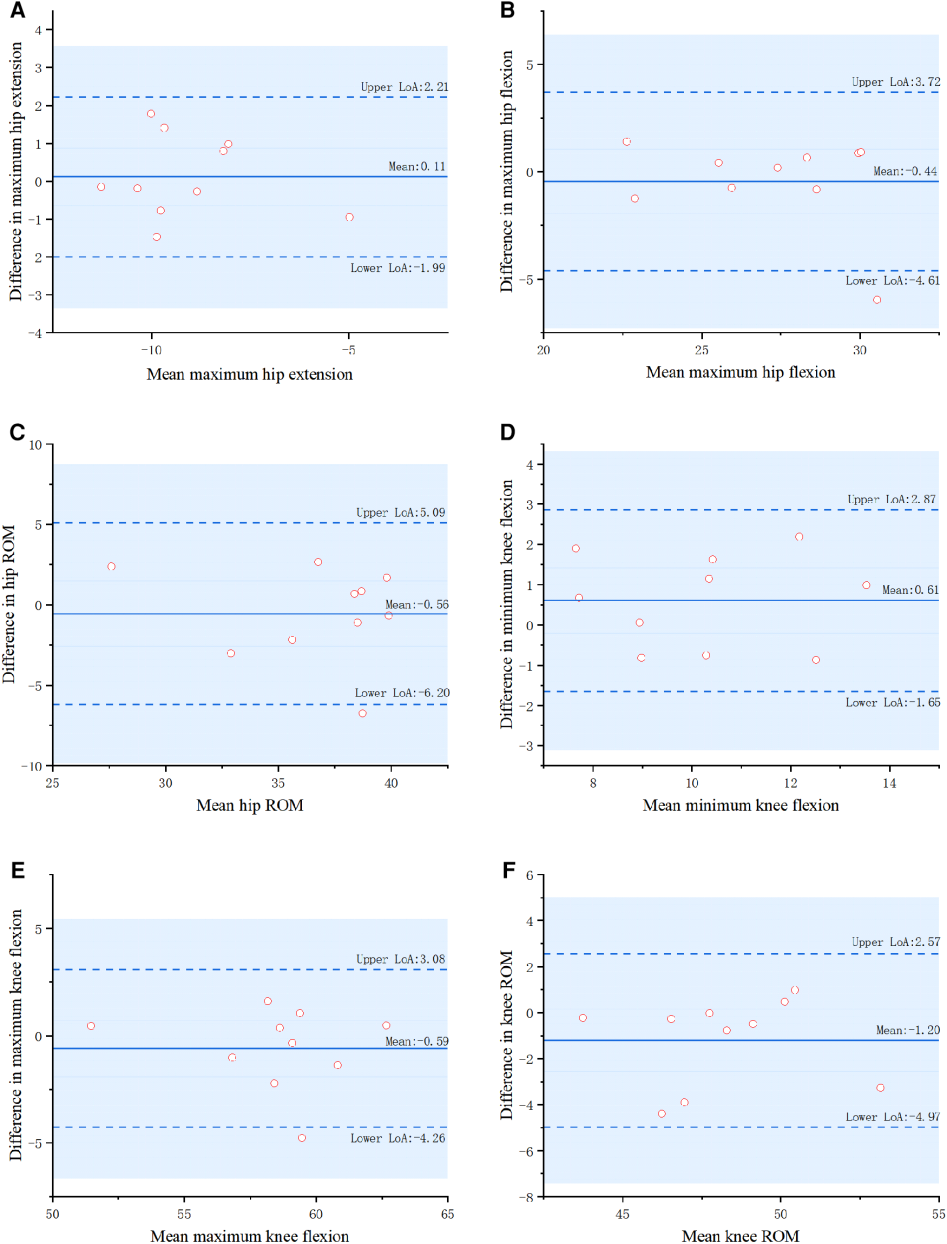
Two methods for parametric measurements of DDH children using Bland–Altman plots (2D ML, 2-dimensional markerless; 3D GA, 3-dimensional gait analysis).

There was no significant difference in gait parameters between the markerless gait evaluation system software and the 3D GA (*P* > 0.05). In terms of relative reliability, the ICC values of all gait parameters ranged from 0.835 to 0.957. The validity reliability was good or excellent. In terms of absolute reliability, the SEM values of all gait parameters were less than 2°, and the reliability was good (Tables 4, 5). The Bland-Altman diagram analysis showed that there was almost no systematic deviation and the consistency was good (Figures 4, 5).

**Table 4:** Sagittal plane mean angles and concurrent validity of TD children for the 2-dimensional markerless (2D ML) method and 3-dimensional gait analysis (3D GA).

Gait variables (°)	2D ML Mean (SD)	3D GA Mean (SD)	*t*-value	*p*-value	ICC	95% ICC	SEM	95% LOA
Maximum hip extension	−12.99 (4.18)	−13.34 (4.32)	1.124	0.277	0.946	0.858–0.980	0.98	−3.27 to 4.29
Maximum hip flexion	30.36 (4.51)	30.63 (4.13)	−1.396	0.181	0.957	0.886–0.984	0.87	−3.54 to 2.75
Hip ROM	43.35 (6.21)	43.95 (5.06)	−1.587	0.131	0.929	0.810–0.973	1.48	−6.51 to 4.42
Minimum knee flexion	7.91 (3.81)	7.46 (3.77)	0.942	0.359	0.921	0.793–0.970	1.05	−3.57 to 4.48
Maximum knee flexion	60.24 (3.36)	61.21 (4.72)	−1.344	0.197	0.835	0.573–0.938	1.65	−6.90 to 4.98
Knee ROM	51.50 (4.94)	53.75 (6.07)	−1.945	0.069	0.903	0.731–0.964	1.71	−7.48 to 4.64

**Table 5:** Sagittal plane mean angles and concurrent validity of DDH children for the 2-dimensional markerless (2D ML) method and 3-dimensional gait analysis (3D GA).

Gait variables (°)	2D ML	3D GA	*t*-value	*p*-value	ICC	95% ICC	SEM	95% LOA
	Mean (SD)	Mean (SD)						
Maximum hip extension	−9.04 (1.75)	−9.15 (1.90)	0.328	0.75	0.905	0.618–0.976	0.55	−1.99 to 2.21
Maximum hip flexion	26.97 (2.75)	27.42 (3.34)	−0.662	0.525	0.863	0.448–0.966	1.11	−4.61 to 3.72
Hip ROM	36.41 (3.85)	36.97 (4.32)	−0.61	0.557	0.859	0.431–0.965	1.5	−6.20 to 5.09
Minimum knee flexion	10.56 (2.09)	9.95 (2.08)	1.67	0.129	0.917	0.666–0.979	0.59	−1.65 to 2.87
Maximum knee flexion	58.20 (2.95)	58.79 (3.20)	−0.994	0.346	0.898	0.588–0.975	0.96	−4.26 to 3.08
Knee ROM	47.64 (2.84)	48.84 (2.77)	−1.97	0.08	0.867	0.463–0.967	1.02	−4.97 to 2.57

## 4. Discussion

This study evaluated the simultaneous validity of a markerless gait assessment method based on the KAPAO algorithm with 3D GA and its test-retest reliability in terms of gait in children. To the best of our knowledge, this is the first quantitative evidence based on human pose estimation algorithms for children's gaits.

Compared to the 3D GA system, the markerless gait evaluation system based on the human posture estimation algorithm is easier to popularize and use, and the characteristic of not marking is more helpful to encourage children's co-operation. Compared with traditional visual qualitative analysis, it has obvious advantages and can provide accurate quantitative evidence for disease diagnosis. In recent years, human pose estimation algorithms have developed rapidly. Osokin et al. (32) proposed a bottom-up method for multi-person attitude estimation based on an open pose. This method has better accuracy and faster operation speed (26 frames/s); Cheng et al. (33) suggested a new bottom-up human posture estimation method—HigherHRNet—which solves the problem of time-scale changes in bottom-up multi-person attitude estimation, locates keypoints more accurately, deals with scale changes more effectively. At present, research on human posture estimation algorithms is more focused on adults, whereas research on children remains limited. Given that children and adult's gaits differ, there is an urgent need to develop a markerless motion capture system that can be applied to children's gait. Accordingly, this study used the open-source KAPAO human posture estimation algorithm (34)—a new single-stage 2D multi-person keypoint and attitude detection method proposed by researchers at the University of Waterloo in Canada that can compensate for some of the defects in the thermal map. In the algorithm verification, we observed that KAPAO is faster and more accurate than other methods for extracting children's gait data. Therefore, we developed a markerless gait evaluation system using the KAPAO human posture estimation algorithm to evaluate children's gait. To ensure the accuracy of children's crowd recognition, we established corresponding gait datasets. Based on this, the reliability and validity of the system were analyzed and the feasibility of its practical clinical application was discussed.

Previous studies have examined the reliability of physiotherapists' analyses of children's gait. Ross et al. (35) used GAITRite trails and single cameras to explore the reliability of hip, knee, and ankle angles in the sagittal plane, including marked and unmarked. In the case of marked planes, experienced physiotherapists showed good to excellent reliability (ICC: 0.77–0.97), and in unmarked case, experienced physiotherapists showed moderate to good reliability (ICC: 0.51–0.86). Saner et al. (25) used a two-dimensional real-time motion tracking method to test whether hip and knee angles in the sagittal plane were consistently measured, which also showed good to excellent reliability (ICC >0.75), and the results of the two measurements were in good agreement. In this study, the markerless motion capture system based on the KAPAO human posture estimation-based algorithm showed moderate to good test-retest reliability (ICC: 0.736–0.894), which is consistent with the results of Ross et al.

Some past reliability studies relied only on the relative coefficient for the evaluation without considering absolute error (31, 36). In contrast, we measured the absolute error and found that the SEM values of all gait parameters were less than 3°, which indicates good absolute reliability, thus providing more comprehensive and useful data. The correlation coefficient of the two measurements of the markerless gait evaluation system software has moderate to good reliability. One possible factor is that the gait repeatability of children is lower than that of adults (37). Stolze et al. (38) tested the spatio-temporal gait parameters of adults and children and demonstrated that the test-retest reliability of children was worse than that of adults and the variability between groups was higher. Sutherland et al. (37) found that children's gait is more likely to vary than that of adults, and the variability decreases with age; therefore, in this study, moderate to good test-retest reliability was an acceptable result for children's gait.

According to the present study's results, the software of the markerless gait evaluation system has good or even excellent validity compared to 3D GA software and can accurately calculate the relevant joint angle gait parameters. Testing revealed that the correlation coefficient of gait parameters measured by the two methods was 0.835–0.957, and all SEM values were less than 3°. At the same time, the Bland–Altman diagram analysis indicated that the results measured by the two methods are in good agreement. Together, these data demonstrate that the markerless gait evaluation system has good accuracy. This study mainly tested whether the software of the markerless gait evaluation system could accurately and reliably measure children's gait parameters, which are currently lacking in research on markerless gait evaluation systems. In the field of markerless gait assessment systems, most studies have focused on adults and children with cerebral palsy (39, 40). Sandau et al. (24) applied their 2DML method on ten healthy adults and found that knee joint kinematics generally overestimated knee flexion and hip joint kinematics by 2.8 ± 1.9 and 0.4 ± 1.5°, respectively, compared with the 3DGA system. Liang et al (41) combined OpenPose and 3DPoseNet markerless pose estimation algorithms to recognize the gait of the elderly with moderate to good reliability and validity. Andrea Castelli et al. (13) proposed a 2D ML technique for sagittal kinematics analysis of the lower extremities using a single camera that tested all joints of adults with a high correlation (0.82 < R2 < 0.99). Evelina et al. (39) tested the reliability of gait parameters in children with cerebral palsy. Compared with the 3DGA, the 2DML method overestimated the knee flexion/extension angle by 3.3–7.0°. The reliabilities of the 2DML and 3DGA were mostly good to excellent. However, there are few studies on TD children or DDH children, and significant differences in gait among different groups. Compared to TD children, children with cerebral palsy have more variability in some kinematic gait variables. Therefore, it is of great significance to design a markerless gait evaluation system for children and to test its reliability and validity.

In addition, it should be noted that when evaluating children's gait, younger children are sometimes more difficult to get to cooperate with procedures and require reasonable guidance from their parents (42). While doing so, parents may be photographed in the video; therefore, accurately extracting the gait parameters of the tested children under the backgrounds of different personnel is critical (18). The system software designed in this study captured and measured the gait parameters of the tested children and was adjusted so as to not be affected by the surrounding environment as much as possible.

This study not only verified the reliability and effectiveness of the markerless gait evaluation system software in TD children, but also verified its reliability and effectiveness in the population of DDH children. The results show that the system software has good reliability and effectiveness in these two kinds of population. From the measurement results, it can be seen that the range of motion of hip joint and knee joint of children with DDH is smaller than that of children with typical development. This parameters can provide quantitative reference data for pediatric orthopaedic surgeons and provide help for disease diagnosis and follow-up.

This study has several limitations. Firstly, the sample size of the test was insufficient, the source was singular, the testers were all TD children and DDH children, the age span of the children was large (3–11 years), and children or patients of other age groups were not tested. In practical applications, we found that the compliance of young children is poor and it is difficult for most children under three years of age to co-operate with the examiner's instructions to carry out corresponding operations (Supplementary Video S3). In the future, we will further improve the method and extend it to more age groups and patients (e.g., those with congenital talipes equinovarus and hemiplegia). Secondly, considering the limited outpatient environment and time, the actual interval between the two tests was too short, which may have led to highly reliable analytical results. In future research, the test scene should be expanded and the test time extended to reduce this factor's impact on the results. Finally, the extracted gait parameters are limited, especially the comparison of temporal and spatial parameters and angle change curves is lacking. In future research, we will explore how to obtain spatio-temporal parameters and angle change curves through the developed markerless motion capture system and test its reliability and validity.

## 5. Conclusion

In this study, based on a human posture estimation algorithm, a low-cost markerless gait evaluation system for children was developed that can quickly and intelligently obtain gait parameters related to human walking using ordinary mobile phone cameras. Such an approach overcomes the shortcomings of traditional gait analysis systems, including high cost, limited site availability, and dependence on markers. The test results showed that the method has good reliability and effectiveness, can accurately calculate the relevant gait evaluation parameters of TD children and DDH children, provides reliable gait analysis results, and can be widely used in routine clinical diagnosis and treatment.

## Data Availability

The original contributions presented in the study are included in the article/**Supplementary Material**, further inquiries can be directed to the corresponding authors.
